# Needs and Expectations for the myNewWay Blended Digital and Face-to-Face Psychotherapy Model of Care for Depression and Anxiety (Part 2): Participatory Design Study including Mental Health Professionals

**DOI:** 10.2196/68789

**Published:** 2025-12-04

**Authors:** Rosemaree Kathleen Miller, Kathleen O'Moore, Katarina Kikas, Julie-Anne Therese Matheson, Alexis Estelle Whitton, Peter Baldwin, Sophie Li, Melissa Black, Laura Kampel, Nicole Cockayne, Fiona Tuttlebee, Caitlin Fraser, Victoria Carr, Kathleen Varghese, Jill Maree Newby

**Affiliations:** 1 Black Dog Institute, Randwick, NSW, Australia; 2 School of Psychology, UNSW Sydney, High Street, Kensington, NSW, 2252, Australia, 61 (02) 9065 9108; 3 Faculty of Medicine & Health, UNSW Sydney, Kensington, NSW, Australia

**Keywords:** depression, anxiety, smartphone, digital intervention, blended care, participatory design

## Abstract

**Background:**

In blended care, digital mental health interventions (DMHIs) integrate with face-to-face psychotherapy provided in person or via telehealth. To incorporate DMHIs into routine care for depression and anxiety, it is important to understand the needs and expectations of mental health professionals for blended DMHIs.

**Objective:**

The study objective was to partner with Australian mental health professionals in the design of a transdiagnostic, cognitive behavioral therapy–based blended model of care for adults experiencing depression and anxiety.

**Methods:**

Participants were Australian health professionals who treat adults with depression and anxiety. The participatory design process included a web-based survey (N=258), one-on-one interviews (N=14), and a 2-part focus group (N=6). Quantitative and qualitative data were collected through the web-based survey. In-depth qualitative feedback from interviews and the 2-part focus group was subjected to reflexive thematic analysis.

**Results:**

Mental health professionals found blended care with face-to-face therapy more acceptable than telehealth and blended care with telehealth, with standalone DMHIs being the least preferred option. The most common ways in which mental health professionals thought a DMHI could integrate with face-to-face psychotherapy included homework completion (129/178, 72.5%), skills practice to support in-session therapy (128/178, 71.9%), and psychoeducation (127/178, 71.3%). Mental health professionals expect the blended DMHI to be easy to use, flexible, protective of client data, and to include evidence-based content from several therapeutic modalities (eg, cognitive behavioral therapy and mindfulness). Other preferences included mental health professionals being able to prescribe specific program modules to their clients, track the treatment progress of clients, and receive alerts if their clients’ symptoms worsened. In terms of implementation, mental health professionals were concerned about the time and effort needed to use blended care. They suggested that ongoing training and support would help mental health professionals implement blended care with their clients. Monitoring client risk and progress via a web-based dashboard and downloadable summaries was also important.

**Conclusions:**

Designing DMHIs that support psychotherapy for adults with depression and anxiety has the potential to increase access to evidence-based treatment. Involving mental health professionals in DMHI design is expected to increase their acceptance of DMHIs and facilitate the integration of these digital products into routine care.

## Introduction

### Background

The global impact of mental disorders continues to grow annually, with significant health, social, and economic consequences [1,2]. Shortages in the mental health workforce and funding for services also impact access to evidence-based care worldwide [3]. Depression and anxiety are two of the most prevalent mental disorders globally, and psychotherapies are currently recommended as first-line treatments for both disorders, either alone or in combination with pharmacotherapies [4,5]. Cognitive behavioral therapy (CBT) has the strongest evidence base for reducing depressive and anxious symptoms, with recent research confirming moderate to large effects on depressive disorders across more than 400 randomized controlled trials (RCTs) [6] and large effects on anxiety symptoms across 66 RCTs [7]. Transdiagnostic psychotherapies are effective for depression and anxiety and are increasingly favored by the clinical community, as these psychotherapies target the shared symptoms, causes, and maintaining factors of co-occurring disorders, such as depression and anxiety [8-11].

### Digital Mental Health Interventions

Unfortunately, most adults who meet diagnostic criteria for depression and anxiety do not access treatment [12,13]. Digital mental health interventions (DMHIs) help to overcome several barriers to accessing treatment (eg, waiting lists, high out-of-pocket costs, and lack of access to skilled mental health practitioners) [3,14]. DMHIs typically include psychotherapy-based content and may be especially beneficial for people who cannot access psychotherapy delivered by a mental health professional. Originally, DMHIs were delivered by computer (eg, CD-ROM) [15,16], but they have now evolved to the point where DMHIs can include artificial intelligence [17]. Today, DMHIs primarily consist of mobile apps and internet-based programs delivered on digital devices, such as smartphones, tablets, laptops, and desktop computers. Self-guided DMHIs enable individuals to self-manage depressive and anxious symptoms with evidence-based skills (eg, CBT) via automated, self-paced modules. Guided DMHIs work similarly but are used with remote support from a health care professional or a paraprofessional, such as a trained coach. Several meta-analyses [16] show that self-guided and guided DMHIs, particularly internet CBT-based programs, significantly improve symptoms of depression (83 studies; Hedge *g*=0.52, 95% CI 0.43‐0.60 [15]) and anxiety (47 studies, Hedge *g*=0.80, 95% CI 0.68‐0.93) [18]. Early evidence suggests that mobile DMHIs could also be used to effectively treat depression and anxiety, albeit with smaller effect sizes than digital CBT delivered via the internet or computer software [19].

Despite decades of research evidence, the uptake of DMHIs into practice is limited due to high dropout rates and poor user engagement of clients, especially in self-guided formats [20,21]. Acceptance of DMHIs by mental health professionals is another important factor influencing their inclusion in routine clinical care, as health professional endorsement can impact whether a client is willing to use a DMHI [20] and if DMHIs are considered a suitable option for referral [22]. Issues that impact DMHI acceptability by mental health professionals include concerns about program credibility [23,24], DMHI accessibility for people with digital, writing, or reading difficulties [22,25], and the appropriateness of DMHIs for people with severe mental health difficulties [26,27]. Moreover, safety concerns about DMHIs are linked to low acceptability and a reduced likelihood that health professionals will incorporate these products into routine practice [14,26,27]. For example, a qualitative study with Australian psychologists (N=10) [26] found that they preferred face-to-face psychotherapy over standalone DMHIs and were concerned that DMHIs increased the risk of severe symptoms being missed (eg, suicidality) and lacked personalization and human connection, especially self-guided DMHIs.

Combining DMHIs with face-to-face psychotherapy (blended care) can address the limitations that affect DMHI acceptability when integrated into routine clinical care [23,25,26]. Blended care extends upon guided DMHIs by using the DMHI to facilitate in-person or telehealth assessment and treatment led by a mental health professional. The DMHI may be combined with psychotherapy within a treatment plan or supplement psychotherapy as an adjunct, either in-session or between sessions. Much of the existing research on blended care has focused on models of care that support mental health professionals who deliver psychotherapy, such as therapists and psychologists. Blended care can be defined or implemented in several ways; however, one commonality between blended approaches is using DMHIs to enhance psychotherapy and ensure that DMHIs are used as effectively as possible. Examples include reinforcing psychoeducation, symptom tracking, or helping clients to practice skills learned in face-to-face therapy sessions. Findings from Germany, the Netherlands, and Denmark indicate that blended care was acceptable to CBT-based therapists and psychologists before and during the COVID-19 pandemic [28-31]. Research conducted before the COVID-19 pandemic also found that blended care is more acceptable to health professionals and care providers than standalone internet treatment for depression across all levels of severity [32].

### Evidence for Blended Care

To date, most research on blended care, especially for depression, was conducted in Europe before the COVID-19 pandemic. Blended care has been tested in several RCTs and pilot trials across Europe in high-income countries, such as Germany, Denmark, Sweden, and the Netherlands, with promising results [25,33-37]. European research also shows that local mental health professionals value blended DMHIs that are flexible to use [38,39], can be customized to suit client needs [28,31,40], and help clients with symptom tracking and homework completion between therapy sessions [28,38]. Adequate training and minimizing the time burden of DMHI use with clients is also important to European mental health professionals [28,31,38-40]. The European research suggests that blended care is scalable and could enhance mental health professionals’ acceptance of DMHI use in routine care, as well as improve client engagement by increasing DMHI uptake and adherence. However, both before and during the COVID-19 pandemic, mixed results were found for blended RCTs outside of Europe in other high-income countries, such as the United States, Japan, and Australia [25,33,41-43]. There is a need to investigate whether the European findings generalize to blended care in non-European countries.

Access to mental health treatment varies worldwide [3], and, by extension, a blended care approach must be tailored to the specific mental health system of a country to function optimally. In the case of Australia, most people with depression and anxiety are provided a mental health treatment plan by their general practitioner, who then refers the person to other health professionals as needed [44]. An Australian with depression or anxiety may receive support from psychiatrists, social workers, or occupational therapists; however, psychotherapy is usually delivered by a psychologist in the private mental health system, with government payments providing partial rebates for up to 10 therapy sessions per calendar year [44,45]. Australian psychologists also currently prioritize CBT due to this therapy being recommended as first-line treatment for depression and anxiety [4,5]. In principle, blended care could work in Australia, as several research studies show the combination of DMHIs with psychotherapy sessions or health professional support may improve symptoms of depression [46-48], anxiety [48], panic disorder [49,50], comorbid depression and alcohol use [51], and hoarding disorder [52]. However, several of these studies have small sample sizes [46,48,52] or no control group for the condition that includes a DMHI combined with psychotherapy or health professional support [47,49,50].

Participatory approaches, including co-design, involve partnering with the intended target audience during the creation of interventions to identify potential issues early and ensure the intervention design reflects their preferences [53,54]. This type of research has been extensively used in DMHI research [54]; however, to date, only 1 participatory [39] design study from Germany has examined the needs and expectations of mental health professionals for blended care using participatory principles. Behr and colleagues [39] consulted with psychotherapists and people with lived experience of mental illness during the COVID-19 pandemic to design a transdiagnostic and transtheoretical internet-based DMHI for blended care. Many of the preferences expressed by psychotherapists aligned with other European blended care studies. However, Behr and colleagues [39] also found that psychotherapists wanted the blended DMHI to protect client data and privacy, contain scientifically sound content, and facilitate the therapeutic relationship with clients. More participatory research on blended care is needed to (1) optimize how the DMHI is deployed to support psychotherapy and (2) ensure the blended care model is adapted to the specific country’s mental health system.

### Rationale, Objectives, and Aims

Prior research on blended care may have used DMHIs that were not designed to support psychotherapy (ie, they are self-guided and do not integrate with psychotherapy). This puts increased pressure on mental health professionals, and the example of Behr and colleagues [39], along with other European research on blended care [38,40], shows that participatory research is needed to ensure that blended DMHIs align with the preferences of mental health professionals and integrate into existing treatment pathways. Moreover, differences between mental health treatment systems could have contributed to mixed results in blended care trials conducted outside Europe [25,33,41-43]. In addition, even in a high-income country like Australia, there is an estimated 32% shortfall in the mental health workforce [55], further highlighting the need to adapt blended care models to specific mental health systems. This study is part of a larger project to create a transdiagnostic DMHI for Australians with depression and anxiety that could be used self-guided or integrated into routine clinical care. Because most Australians with depression and anxiety are treated in the private mental health system [44], it is currently unknown (1) if blended care would be acceptable to Australian mental health professionals, (2) what their preferences for blended care are, and (3) if blended care would work with the current government reimbursement model for psychotherapy sessions.

To our knowledge, this will be the first Australian study to use participatory design principles to design a new CBT-based blended care model for depression and anxiety with mental health professional input. Phase 1 involves 2 multistage participatory design studies that include web-based surveys, qualitative interviews, and a 2-part focus group with mental health professionals and Australian adults with lived and living experiences of depression and anxiety. Results from the participatory design study with people with lived experience are reported separately in Part 1 of this manuscript series [56]. For mental health professionals, our main aim was to determine their needs and expectations for a DMHI to be used with their clients in blended care. The present findings will inform the creation of a DMHI that addresses clients’ needs [56], is acceptable to mental health professionals, and can be integrated into routine treatment for depression and anxiety as part of a CBT-based blended model of care. A secondary aim was to identify factors affecting the training, support, and resources that mental health professionals would need to implement the DMHI into their clinical practice.

## Methods

### Participatory Design Approach

A mixed methods convergent parallel design [57] was used, which included a web-based survey, qualitative interviews, and a 2-part focus group with a convenience sample of mental health professionals. Throughout data collection, participants were encouraged to discuss any DMHIs they used in practice, not just those created by the Black Dog Institute. Stage 1 involved a web-based survey designed to capture the perspectives of a diverse range of Australian health professionals who provide mental health care to adults with depression or anxiety in their clinical practice (eg, general practitioner, mental health nurse, social worker, psychologist, and psychiatrist). The web-based survey was created in line with the Checklist for Reporting Results of Internet E-Surveys [58]. In stage 2, a subset of mental health professionals participated in one-on-one web-based qualitative interviews to collect more focused data to address the study aims and conclusions [57]. Finally, in stage 3, a small sample of psychologists was recruited for a 2-part focus group to provide more targeted feedback on implementing the new blended care model. Five psychologists external to the research team served as health professional advisors throughout stages 1-3. The health professional advisors were consulted regularly throughout each stage to confirm and double-check the findings, and all 5 participated in a 2-part focus group for the study. To further mitigate potential bias, the core research team also worked with a diverse range of nonresearchers, including user experience, IT development, innovation, and service delivery professionals before, during, and after data collection. Questions for the web-based survey, interview, and focus group were developed via collaboration between the core research team, health professional advisors, and user experience experts.

### Ethical Considerations

Ethical approval was received from the University of New South Wales Human Research Ethics Committee (HC200541). All mental health professionals provided informed consent for the use of their data for research purposes. If participants wished to withdraw their consent, they could contact the research team. All participant data from the web-based survey, interviews, and focus group sessions were deidentified, recoded, and stored in a password-protected file only accessible to the research team. Participants could enter a prize draw to win an Aus $100 (US $65) online gift card for completing the web-based survey. A prize draw was selected as the reimbursement method to minimize the likelihood that a participant would complete the web-based survey solely for compensation. Survey respondents were randomly invited to complete one-on-one interviews and were reimbursed with an Aus $30 (US $20) online gift card. Due to the high level of group interactivity required for a 2-part focus group compared to the interviews, participants were reimbursed with an Aus $200 (US $130) online gift card for attending both 1-hour sessions.

### Stage 1: Web-Based Survey

The web-based open survey took approximately 20 minutes and was created with Qualtrics software (version 2020; SAP Inc). Eligibility criteria for health professionals included (1) that they provide mental health care to adults with depression or anxiety, (2) were 18 years of age or older, (3) were fluent in English and (4) were currently living in Australia. The web-based survey was advertised between October and December 2020 via the Black Dog Institute website, the Black Dog Institute health professional network, social media (eg, Facebook [Meta Platforms Inc] and Instagram [Meta Platforms Inc]), existing professional organizations, and peak clinical bodies for mental health professionals (eg, the Australian Psychological Society). To participate, health professionals were directed to the Black Dog Institute website to access a web link for the Qualtrics (2020) survey. The web-based survey began with a participant information sheet and consent form, which included details on the purpose of the research study, eligibility criteria, reimbursement, participant data storage, and the estimated time required to complete the survey. The web-based survey included questions on participants’ demographics, clinical practice details, past and current use of DMHIs, and their needs and expectations for a blended DMHI (refer to Multimedia Appendix 1). The web-based survey also included 2 questions from Topooco et al [32] focused on the challenges health care professionals and clients face when integrating digital technology into psychotherapeutic treatment for depression and anxiety (also refer to Multimedia Appendix 1).

### Stage 2: Interviews

A subset of survey respondents was randomly invited via email to participate in one-on-one interviews between November 2020 and March 2021. Interviews were conducted until data saturation was achieved. The interviews explored mental health professionals’ attitudes toward DMHIs and their views on blending these programs into routine clinical care. Apart from their participation in the web-based survey, there were no pre-existing relationships between participants and the researchers. Interviews were run online using Zoom videoconferencing software (Zoom Communications Inc), and lasted, on average, 53 (SD 12) minutes. The interviews were semistructured and followed a script developed by the research team based on relevant literature and findings from the web-based survey. No personal details about the researchers were disclosed to participants apart from their involvement in the overall project. Interviews were facilitated by either KO or FT. Open-ended questions were used to prompt discussion during the interview (refer to Multimedia Appendix 1). All interviews were audio-recorded and later transcribed by KK.

### Stage 3: Focus Group

A 2-part focus group was conducted online using Zoom videoconferencing software, with each session lasting approximately 1 hour. The eligibility criteria were the same as those for the web-based survey. However, only psychologists were included, as they were the most common type of mental health professional recruited for the web-based survey and interviews. A 2-part focus group was advertised via email in July 2021, and the 2 sessions were run in July and August 2021, respectively. A convenience sample of 5 health professional advisors and 2 psychologists from the Black Dog Institute psychology clinic was recruited. The researchers were familiar with 7 participants because they worked at the same workplace, but there were no pre-existing personal relationships. One psychologist’s data was excluded from data analysis as they could only attend one of the two focus group sessions. To minimize bias, the sessions were designed by service delivery and user experience professionals without clinical training (FT, CF, and VC) with input from the research team. Session 1 was led by CF, while Session 2 was led by KO, FT, and VC. KK attended both sessions as the note-taker. The focus group script (refer to Multimedia Appendix 1) included questions on factors affecting the implementation of the new blended care model, such as how the psychologists would use blended care with therapy sessions, the training and support needed to implement blended care, and the features to include in an online program dashboard designed for health care professionals as part of the blended care system. Both sessions were audio-recorded and later transcribed by author KO.

### Reflexivity Statement

To reduce potential biases, the core group of researchers involved in thematic analysis included 2 clinical psychologists who were also researchers (JMN and KO) and 3 nonclinician researchers (RKM, KK, and JATM). 2 service design professionals external to the research team assisted with qualitative data collection (FT and CF). 4 members of the core research team had prior experience with reflexive thematic analysis (JMN, KO, RKM, and KK). The 2 clinicians (JMN and KO) had experience in psychotherapy and digital mental health treatment for individuals with clinical depression and anxiety. All 3 nonclinician researchers (RKM, KK, and JATM) had experience with digital mental health research. One nonclinician researcher (RKM) also had experience working with staff and health professionals from substance use and mental health services. All coauthors reviewed the final coding framework for the thematic analysis conducted for the interviews and the 2-part focus group. This included 4 clinical psychologists who were also researchers (SL, MB, PB, and LK), a psychologist who was also a researcher (AEW), 2 user experience professionals (VC and KV), 2 service design professionals (FT and CF), and a public health researcher (NC). We acknowledge that our past clinical and research experiences, as well as our familiarity with the literature, may have introduced biases and assumptions that inadvertently influenced the thematic analysis of the interview and focus group transcripts. The core research team minimized this with regular meetings to discuss the data to ensure transparency and check that our themes and subthemes were grounded in the collected data.

### Data Analysis

Descriptive statistics were performed with SPSS software (version 26.0; IBM Corp). For the web-based survey results, descriptive statistics were calculated based on the actual number of participants who responded to a specific question. Reflexive thematic analysis was selected to examine qualitative data from the one-on-one interviews and a 2-part focus group, as this method was appropriate for answering the research questions [59]. For the interviews, KK cleaned and checked the transcripts against the original audio after each interview and focus group session. Authors (KK, KO, and RKM) then read the interview transcripts several times and, after familiarization, used a general inductive approach to generate the initial set of codes. These codes were then categorized using Microsoft Excel to create the initial coding framework. The coding framework was revised iteratively by authors KK, RKM, and JATM until consensus on themes and subthemes was achieved. All disagreements were resolved through discussion with the research team (including KO, JMN, and AEW) until consensus was reached. The same approach was followed to analyze the focus group transcripts. The transcripts were read several times by KO and RKM, who generated the initial set of codes and then revised the coding framework iteratively with JMN.

## Results

### Stage 1: Web-Based Survey

#### Demographics

Of the 258 participants who provided consent, 157 (60.9%) completed the web-based survey. The average age was 47 (SD 13.74; range 18‐78) years, and most participants were women (n=200, 77.5%), spoke English as a first language (n=227, 88%), and lived in a major Australian city (n=175, 73.8%; Table 1). Statistical comparisons revealed no significant differences in the demographics of participants who completed the full survey versus those who did not (see Multimedia Appendix 2).

**Table 1.** Sample characteristics for the web-based survey.

Demographic characteristics	Participants, n (%)
Gender (N=258)	
Women	200 (77.5)
Men	58 (22.5)
English primary language at home (N=258)	227 (88)
First Nations Australian origin (N=258)	4 (1.6)
Residential location in Australia (N=258)	
Major cities	175 (73.8)
Regional or remote	62 (26.2)
Current role^a^ (N=239)	
Registered psychologist	45 (18.8)
General practitioner	43 (18)
Clinical psychologist	37 (15.5)
Social worker	28 (11.7)
Mental health nurse	26 (10.9)
Counselor	20 (8.4)
Trainee psychologist	11 (4.6)
Psychiatrist	5 (2.1)
Other current role^b^	36 (15.1)
Current work setting^a^ (N=239)	
Private practice	119 (49.8)
Not-for-profit organization	49 (20.5)
Outpatient	38 (15.9)
Inpatient	28 (11.7)
University	25 (10.5)
Other work setting^c^	29 (12.1)

a Percentages do not add up to 100% as respondents were allowed multiple responses.

b Other current roles included occupational therapist, peer support roles, and other types of nurses (apart from mental health).

c Other current work settings included government, hospitals, justice settings, and schools.

#### Practice Details

A total of 239 participants provided data on their current work role and work setting. The most common work roles were registered psychologists (n=45, 18.8%), general practitioners (n=43, 18%), and clinical psychologists (n=37, 15.5%; Table 1). The percentage of different work roles and work settings was largely consistent among participants who did or did not complete the full survey (refer to Multimedia Appendix 2). Participants mostly worked in private practice (n=119, 49.8%), not-for-profit organizations (n=49, 20.5%), and outpatient settings (n=38, 15.9%; Table 1). Across 239 participants, most (n=127, 53.1%) had 10 or more years of experience as mental health professionals, with an average of 15 (SD 11.89; range 0-49) years. Table 2 shows that the most common mental health conditions treated by participants were anxiety (199/221, 90%), depression (190/221, 86.1%), and trauma and stress-related disorders (152/221, 68.8%). The most common psychotherapies used to treat depression and anxiety were CBT (178/216, 82.4%), acceptance and commitment therapy (116/216, 53.7%), and mindfulness-based cognitive therapy (115/216, 53.2%; Table 2).

**Table 2.** Counts and frequency percentages of the most prevalent mental health issues treated by mental health professionals and the most used treatment modalities when working with adults experiencing depression and anxiety.

Practice details	Participants, n (%)
Most prevalent mental health issues^a^ (N=221)	
Anxiety disorders	199 (90)
Depressive disorders	190 (86)
Trauma- and stressor-related disorders	152 (68.8)
Personality disorders	97 (43.9)
Substance-related and addictive disorders	80 (36.2)
Bipolar and related disorders	80 (36.2)
Schizophrenia spectrum and other psychotic disorders	55 (24.9)
Obsessive-compulsive and related disorders	51 (23.1)
Feeding and eating disorders	35 (15.8)
Most frequent treatment modalities^a^ (N=216)	
Cognitive behavioral therapy	178 (82.4)
Acceptance and commitment therapy	116 (53.7)
Mindfulness-based cognitive therapy	115 (53.2)
Solution focused and brief therapy	82 (38)
Dialectical behavior therapy	64 (29.6)
Interpersonal psychotherapy	39 (18.1)
Schema therapy	38 (17.6)
Family therapy	26 (12)
Humanistic approach	23 (10.6)
Couples therapy	20 (9.3)
Psychodynamic approach	19 (8.8)
Eye movement desensitization and reprocessing	12 (5.6)
Psychoanalysis	11 (5.1)
Gestalt approach	10 (4.6)
Jungian approach	6 (2.8)
Other treatment modalities^b^	20 (9.3)

a Percentages do not add up to 100% as respondents were allowed multiple responses.

b Other common treatment modalities included peer support, physical activity, lifestyle advice, acupuncture, and hypnosis.

#### Use of Digital Mental Health in Practice

**Figure 1.**
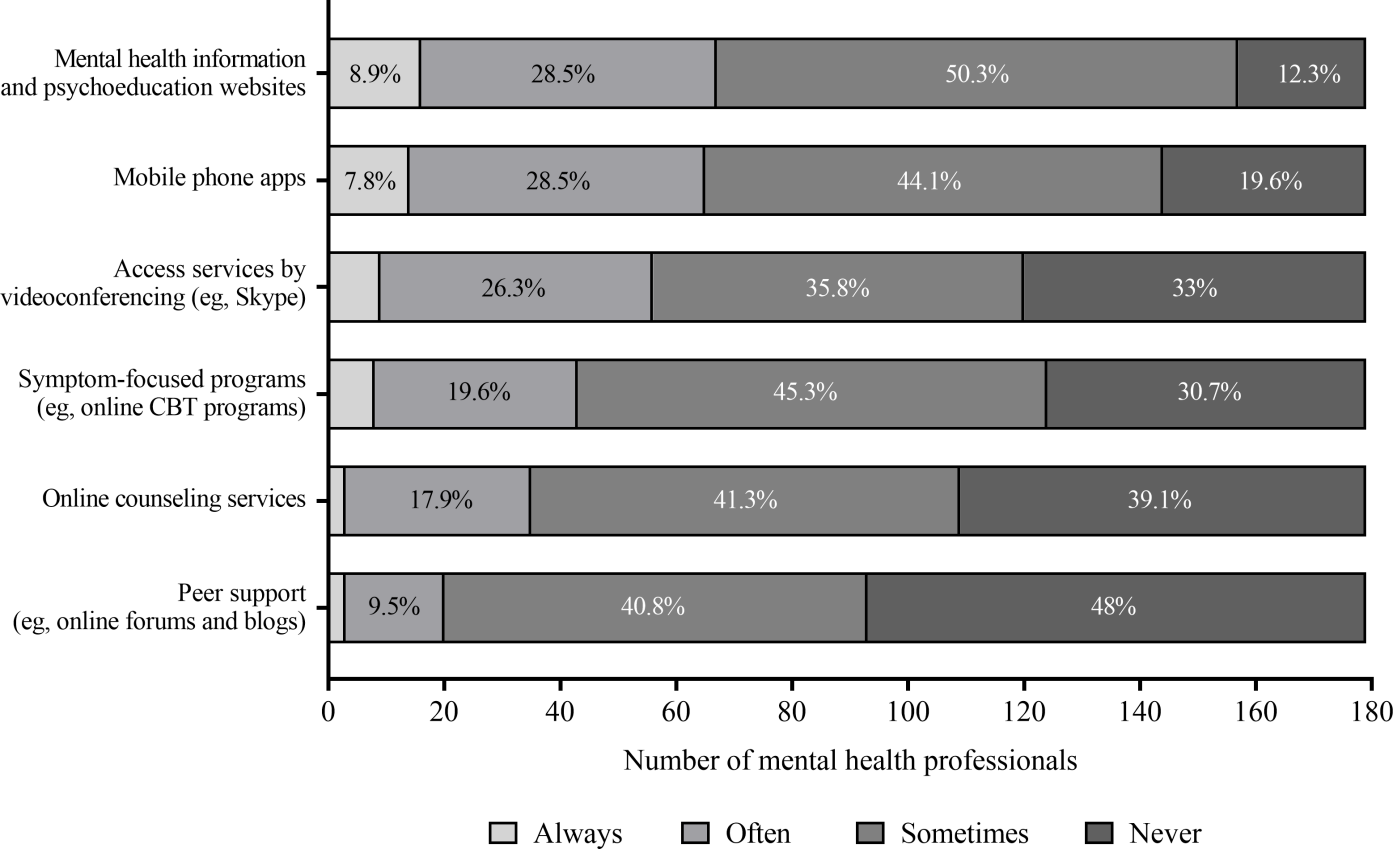
Counts of what type of digital mental health interventions were referred to clients in participants’ clinical practice (N=179). CBT: cognitive behavioral therapy.

Figure 1 (data labels show the frequency percentage) shows how often particpants recommended different types of DMHIs to clients (see Multimedia Appendix 3 for counts). Participants were most likely to always or often refer clients to mental health information and psychoeducation websites (67/179, 37.4%), mobile phone apps (65/179, 36.3%), and videoconferencing services (eg, Skype [Microsoft], Zoom [Zoom Communications Inc]; 56/179, 31.3%). The 3 most common DMHIs that participants would suggest to clients with depression and anxiety were This Way Up (Clinical Research Unit for Anxiety and Depression at St Vincent's Hospital and the University of New South Wales), Smiling Mind (Smiling Mind Pty Ltd), and Headspace (Headspace Health).

#### Is There a Need for Digital Mental Health in Routine Care?

Most participants (135/178, 75.8%) reported a need for an online mental health program that could be integrated into routine care. Among the remaining 43 participants, 9 (5.1%) disagreed and 34 (19.1%) were unsure.

#### Digital Mental Health Preferences

##### Routine Care

Table 3 shows that participants preferred integrating DMHIs into routine care for depression and anxiety by referring clients to homework exercises to do in between sessions (129/178, 71.3%), specific modules to support skills learned in therapy (128/178, 71.9%), and psychoeducation (127/178, 76%). Other commonly preferred ways of integrating DMHIs into routine care included using DMHIs to assist clients with relapse prevention (117/178, 65.7%), tracking symptom changes over time (116/178, 65.2%), and supporting clients who had run out of sessions and could not afford to pay (113/178, 63.5%; Table 3).

**Table 3.** Counts and frequency percentages for integrating DMHIs into routine care, challenges faced by health care professionals and clients when digital technology is integrated with psychotherapy, and factors affecting whether blended care is suitable for a client.

Digital mental health preferences, challenges, and client factors	Participants, n (%)
Ways the program could integrate into routine care^a^ (N=178)	
Refer client to homework exercises	129 (72.5)
Refer client to specific program modules	128 (71.9)
Psychoeducation	127 (71.3)
Relapse prevention once treatment is complete	117 (65.7)
Assessments to track symptoms	116 (65.2)
Support clients who run out of sessions and cannot afford to pay	113 (63.5)
Use in-session to explain or practice skills	107 (60.1)
As a prequel to therapy	88 (49.4)
Challenges for health care professionals^a^ (N=158)	
Low motivation because of critical attitude toward digital technology	73 (46.5)
Not skilled in digital technology	72 (45.9)
Difficulties adapting treatment methods to digital technology	70 (44.6)
Low clinical effectiveness	57 (36.3)
Adherence of the health care professional is low	45 (28.7)
Degree of accessibility and cost of internet	41 (26.1)
Use of digital technology is too time-consuming	32 (20.4)
Other challenges	26 (16.6)
Challenges for clients^a^ (N=158)	
Difficulties with applying self-help strategies	73 (46.5)
Not skilled in digital technology	72 (45.9)
Low client adherence	70 (44.6)
Low motivation because of critical attitude towards digital technology	57 (36.3)
Degree of accessibility and cost of internet	45 (28.7)
Low clinical effectiveness	41 (26.1)
Use of digital technology is too time-consuming	32 (20.4)
Other challenges	26 (16.6)
Client factors for blended care^a^ (N=157)	
Access to a computer, tablet, or phone	128 (81.5)
Online modules relevant to main symptoms or diagnosis of patient	126 (80.3)
Access to the internet	123 (78.3)
Intelligence and cognitive functioning of client	118 (75.2)
Access to a private, safe place to complete program	112 (71.3)
Competence in using digital technology	111 (70.7)
Absence of severe suicidality	109 (69.4)
Absence of psychotic symptoms	100 (63.7)
Absence of acute medical need that hinders client’s ability to participate in treatment	94 (59.9)
Absence of severe substance use	82 (52.2)
Other factors	22 (14)

a Percentages do not add up to 100% as respondents were allowed multiple responses.

##### Program Inclusions

Of the 178 participants, 142 (79.8%) described what they wanted to be included in a blended DMHI. Several suggested evidence-based content from common therapeutic modalities, such as CBT, acceptance and commitment therapy, dialectical behavior therapy, and mindfulness. Participants were also keen for the DMHI to support face-to-face therapy, for example, “flexibility to ’build’ your own exercises with clients or tailor them.” Supporting clients with skills practice and symptom monitoring was also important, for example, “validated assessment measures”, and “sleep tracking and thought monitoring.”

### Challenges of Digital Mental Health

The most common challenges identified by participants for health care professionals were low motivation due to negative attitudes toward digital technology (73/158, 46.5%), not being skilled in digital technology use (72/158, 45.9%), and difficulties with adapting treatment methods to digital technology (70/158, 44.6%; Table 3). The 3 top challenges identified for clients were difficulties with applying self-help strategies (73/158, 64.6%), not being skilled in digital technology use (72/158, 63.9%), and low client adherence (70/158, 62%; Table 3).

### Blended Care Approaches to Treatment

#### Client Factors

The most important factors that participants thought would affect the suitability of blended care for a client were access to a computer, tablet, or phone (128/157, 81.5%), modules relevant to the client’s main symptoms and diagnosis (126/157, 80.3%), internet access (123/157, 78.3%) and the client’s intelligence and cognitive functioning (118/157, 75.2%; Table 3). Other important client factors included access to a private, safe space to use the DMHI (112/157, 71.3%), and competence in DMHI use (111/157, 70.7%). Most participants considered the absence of severe suicidality (109/157, 69.4%), psychotic symptoms (100/157, 63.4%), acute medical needs affecting the client’s treatment (94/157, 59.9%), and severe substance use (82/157, 52.2%) to be important client factors for blended care as well.

#### Acceptability

Participants (N=157) rated acceptability with a 100-point scale (0=Not at all, 100=Extremely acceptable) for (1) standalone digital programs, (2) telehealth sessions, (3) blended care with digital programs and face-to-face sessions, and (4) blended care with digital programs and telehealth sessions. Participants found blended care with digital programs and face-to-face sessions the most acceptable treatment option (mean 71.61, SD 25.48), followed by telehealth sessions (mean 68.38, SD 23.72) and blended care with digital programs and telehealth sessions (mean 64.65, SD 25.86). Standalone digital programs were the least acceptable option to participants (mean 38.81, SD 25.86).

#### Sharing of Client Information

Most participants preferred client information to be shared with them via a secure email account (99/157, 63.1%) or sent to a secure web portal where they could log in and see the information (91/157, 58%). A minority wanted their client’s information shared with them via SMS text messaging to a nominated phone number (15/157, 9.6%) or through another information sharing method (16/157, 10.2%), such as a printable report or via an existing practice management system. Some participants were not sure (14/157, 8.9%) or did not mind (11/157, 7%) how client information was shared with them. The types of client information that participants wanted to share and receive from the blended DMHI included program adherence and completion, symptom tracking with validated and subjective (eg, sleep and mood) measures, and client feedback on treatment (eg, benefits, difficulties, or questions they have). The preferred time interval between receiving client information from the DMHI varied. Most participants preferred a weekly or fortnightly interval, and others wanted to tailor the interval to the client or treatment timeline, for example, *“*every week to begin with, moving to larger intervals as treatment progresses*.”*

### Stage 2: Interviews

#### Demographics

A total of 14 mental health professionals participated in the one-on-one interviews. Their mean age was 46 (SD 13; range 26‐73) years. Half of the participants were female (7/14; 50%), and the other half were male (7/14; 50%). Overall, 11/14 (79%) participants lived in major Australian cities, and 3/14 (21%) lived in regional or remote areas of Australia. The sample consisted of registered psychologists (5/14, 36%), clinical psychologists (5/14, 36%), mental health nurses (3/14, 21%), and a counselor (1/14, 7%). Participants had worked as mental health professionals for an average of 16 (SD 9.95; range 4‐35) years. Participants worked in private practice (5/14, 36%), outpatient settings (4/14, 29%), universities (4/14, 29%), and not-for-profit organizations (2/14, 14%).

#### Thematic Analysis Findings

##### Overview of Findings

Six key themes were identified related to blended care for depression and anxiety: (1) blended care preferences, (2) promote client engagement, (3) prioritize client safety and duty of care, (4) minimize burden on time-poor clinicians, (5) program is credible and trustworthy, and (6) client barriers to using program. Illustrative quotes for each subtheme within the 6 themes are provided in Table 4.

**Table 4.** Thematic analysis themes and subthemes with example quotes.

Themes and subthemes	Example quotes
1. Blended Care Preferences	
Supports Skills Practice, Psychoeducation, and Lifestyle Changes	“If we’re able to provide them with resources, [like] psychoeducation material...alongside the therapy that we’re doing, it increases the support provided to the client.” [Participant 71]
Helps Clinician Monitor Treatment Progress	“...I also want to know whether they’re finding therapy effective and helpful and what they find helpful and what they don’t find helpful.” [Participant 14]
Prescribe Program Modules to Clients	“In a perfect world, a way to digitally prescribe it for people to track their progress, and then to be able to facilitate...not just like the way they engage with it, but in session tools.” [Participant 15]
Walk Through Program Content in Session	“My preference...is to work through a program with a client, to have some oversight on what they’re doing, to review the content with them, to answer any questions that they have.” [Participant 8]
Trial the Program from the Client’s Perspective	“If I were to give it to somebody or lead someone to it, I would want to do it myself, first of course.” [Participant 3]
2. Promote Client Engagement	
User-Friendly and Inviting Interface, With Minimal Text	“I like that it’s very simple and easy, a lot of the communication around concepts is done visually and in videos, there’s no long slabs of text.” [Participant 15]
Flexible with Lots of Variety	“It’s important to have lots of different...ways of delivering information. So, whether it be...printed, video, audio and animated or something like that...it kind of keeps your interest and it’s kind of interesting and stimulating, you’re not just reading realms of stuff.” [Participant 12]
Program Content Adjusts to Meet Client Needs	“The other thing that I think would be really, really helpful is if there was some way that you know this online program is personalized...because I think sometimes if you mention a whole lot of stuff, you know, clients find it a little bit trite, you know, like eat well and you know look after yourself. It’s not really meaningful to them.” [Participant 14]
Interactive Activities and Features	“...a bit interactive with a quiz or a little video or somebody talking about their experience, you know, that’s just short and sweet, you know, a three-minute video and practical things.” [Participant 24]
Relatable and Encouraging Stories Shared by Real People	“...having stories where people share their story, that can be very powerful.” [Participant 48]
3. Prioritize Client Safety and Duty of Care	
Directs Clients to Crisis Support When Needed	“If there [were]...links to crisis services that are available and perhaps, you know, if people are screening as really high, knowing that they’re going to get some kind of automated message to remind them to seek support immediately...that’s really important.” [Participant 9]
Helps Monitor Client Safety	“Hmm, if they are feeling worse...maybe that’s the possibility of the clinician being alerted if their scores are really low...then the clinician could do a follow-up call, or like a check-in.” [Participant 12]
Supports Maintenance of Professional Boundaries	“...we’ll organize to call [the client] or send a text or we’ll organize for a crisis service to ring or...and you work out with them what, you know, that’s a bit of a negotiation process about what they’d be comfortable with and what you’re obliged to do under your duty of care.” [Participant 39]
Not Suitable for Clients with Severe Mental Distress	“With the population I work with, no, I mean if I was working with a more severe population, I might be a little bit less willing to use online programs without more support.” [Participant 10]
4. Minimize Burden on Time-Poor Clinicians	
Quick Reference Guides and Resources	“So, I think first of all, helping clinicians have a quick and easy way of understanding what the content of a module or whatever it is, so they know if it’s appropriate.” [Participant 15]
Integrates with Existing Client Management Systems	“I don’t know if it’s going to come into it, but also not being easily able to refer like say, for example, some of our clinicians they’ve got electronic medical records and databases already set up like if there was an easy trigger in those systems to kind of alert someone to refer to another service.” [Participant 83]
Web-Based Program Dashboard	“If there is a clinician dashboard kind of interface, being able to have access to that, so I can see before the session, ‘okay, great, they have completed this task.’” [Participant 8]
5. Program Is Credible and Trustworthy	
Evidence-Based and Reputable	“I’d want it to be, you know, peer-reviewed...and it’s gone through all the you know research, efficacy, validity, all that sort of stuff, so I’d want it to have all that as the foundation.” [Participant 33]
Secure and Protects Client Privacy	“...maybe a button with ‘would you like your clinician to be able to see this?’, ‘yes or no’, something that they can control, so they can say yes, just before the session and then after the session, they can hide it again if they don’t want the clinician to have ongoing access.” [Participant 10]
6. Client Barriers to Using Program	
Program Cost	“I think once you put a cost on it, you can lose a lot of people.” [Participant 24]
Digital Interventions Not Appropriate for Some Clients	“The ability to engage with the app and take on board information that’s there with the reading and the content requires a level of cognitive functioning and, you know, self-awareness and conscious thought. If all those are getting scrambled for you, then this isn’t going to work.” [Participant 62]

##### Theme 1: Blended Care Preferences

###### Theme Overview

This theme refers to how mental health professionals envisioned the blended care program would work for clients receiving routine clinical care for depression and anxiety.

###### Supports Skills Practice, Psychoeducation, and Lifestyle Changes

Participants emphasized the importance of modules in the program supporting skills taught in face-to-face therapy, such as psychoeducation and healthy lifestyle content (eg, sleep hygiene, physical exercise, and nutrition). Including this content would allow clinicians to assign psychoeducation and skills practice activities to clients between sessions, freeing up therapy sessions for more challenging and tailored skill development.

###### Helps Clinician Monitor Treatment Progress

Several participants wanted to track whether clients found their therapy sessions helpful and monitor the severity of client symptoms across sessions to indicate progress. Participants suggested including measures for client-reported outcomes and experiences in the program (eg, outcome rating scale and session rating scale). Some participants were also interested in viewing which modules and activities clients had completed in the program.

###### Prescribe Program Modules to Clients

Participants preferred to prescribe specific modules or activities in the program so that their clients were assigned content and activities relevant to their needs or which assisted clients in practicing a skill they were currently learning with their therapist.

###### Walk Through Program Content in Session

When using a DMHI, participants wanted to guide clients through the content during therapy sessions to help familiarize them with the program and help clients feel more comfortable using it. Participants also thought that demonstrating how the program worked to their clients would address practical barriers, such as client reporting of technical issues.

###### Trial the Program From the Client’s Perspective

Participants preferred to trial the program from the client’s perspective before recommending it. This feature would allow participants to verify the program’s quality, ease of use, and content before recommending the program to clients.

### Theme 2: Promote Client Engagement

#### Theme Overview

User engagement with the blended care program was an important consideration for interviewees. Theme 2 includes the features, functionality, and content within a DMHI that mental health professionals suggested would enhance client engagement with the program.

#### User-Friendly and Inviting Interface With Minimal Text

Most participants believed that the program should be easy and intuitive for clients to use and stay engaged with. Some participants suggested that the onboarding experience for clients should be simple and include minimal registration questions. Other suggestions included using clear and friendly language to explain concepts and avoid *“*long slabs of text.” It was also important that the program was designed with an aesthetically pleasing interface (eg, colorful, comic-style graphics and animations).

#### Flexible With Lots of Variety

Many participants thought clients would stay engaged with the DMHI if it was flexible, modular, and included a range of content, options, and functionality. Participants also believed clients would like to choose which module or activity they wanted to complete rather than having one suggested. Some participants thought that transdiagnostic content would be more engaging for clients than disorder-specific content and recommended including transdiagnostic modules that address multiple concerns common for people with anxiety and depression (eg, perfectionism, rumination, self-esteem, and procrastination).

#### Program Content Adjusts to Meet Client Needs

Some participants thought content personalized to clients’ needs would help them stay engaged with the program. For example, if a client indicated they wanted to improve their relationships with others or manage procrastination, the program would show information or modules relevant to these topics in response.

#### Interactive Activities and Features

Several participants believed interactive activities and features would enhance client engagement, such as practical activities that require users’ active input, such as quizzes and chatbots. Other suggestions included videos, audio tracks, diagramming tools, and activities, such as behavioral activation or exposure stepladders where their client could input content relevant to them.

#### Relatable and Encouraging Stories Shared by Real People

Some participants believed that incorporating other people’s experiences of mental ill health and treatment into the program would enhance client engagement. These participants thought a video would be a good way to include these stories and that the examples should be relevant and encouraging for clients.

### Theme 3: Prioritize Client Safety and Duty of Care

#### Theme Overview

This theme focuses on participant perspectives about the importance of prioritizing and safeguarding client safety while also assisting mental health professionals in setting clear boundaries around their duty of care to clients.

#### Directs Clients to Crisis Support When Needed

A common concern was ensuring clients could access crisis support if they were feeling unwell or experiencing suicidal ideation while using the DMHI. Most participants wanted contact details of emergency and mental health support services included (eg, Lifeline and Suicide Call Back Line), while others endorsed a more personalized approach where a user input (eg, endorsing suicidal thinking) would trigger messages or risk alerts, such as advising the client to seek help immediately and then directing them to crisis support information.

#### Helps Monitor Client Safety

Participants wanted the program to help monitor client safety and alert the clinician if their client’s symptoms worsened or they endorsed indicators of elevated suicide risk. A common suggestion was to use validated questionnaires to flag if a client’s symptoms were worsening. Some participants thought the DMHI could prompt mental health professionals to check in with their clients or reassess their treatment plans.

#### Supports Maintenance of Professional Boundaries

Participants expressed concern about their increased duty of care between therapy sessions when using a DMHI with clients (eg, being required to contact clients at risk outside of sessions). Some also thought the program could help mental health professionals manage their duty of care and client safety. This included (1) guiding discussions about duty of care with clients, (2) setting boundaries around the mental health professional’s availability, and (3) clarifying which client information would be shared with mental health professionals. Participants thought that this could be facilitated through the onboarding content of the DMHI and automatic notifications about elevated client risk to mental health professionals.

#### Not Suitable for Clients With Severe Mental Distress

Some participants felt a DMHI, even when used with blended care, would not be appropriate for high-risk clients or those experiencing severe distress. Examples included clients who were suicidal, experiencing significant challenges with perceptions of reality (eg, psychosis and personality disorder) and those with severe distress or functional impairment (eg, severe depression).

### Theme 4: Minimize Burden on Time-Poor Clinicians

#### Theme Overview

This theme describes how DMHIs can reduce the burden on mental health professionals who may already be short of time.

#### Quick Reference Guides and Resources

Many participants thought simple, quick, easy-to-use guides and resources would help them decide whether the DMHI was appropriate for clients. Several participants specified that short overviews of content or program modules, the program structure, and details to pass on to the client (eg, time commitment) would be helpful. The preferred medium for quick reference guides and resources was varied and included written summaries, short videos less than 2 minutes long, booklets, and brochures.

#### Integrates With Existing Client Management Systems

Some participants wanted the blended DMHI to integrate with existing software and client management systems used in their practices. Examples included sending questionnaires or referring clients to the program digitally via client management systems that mental health professionals already had in place to avoid the need for additional record-keeping and the risk of duplicate records.

#### Web-Based Program Dashboard

Several participants wanted access to a dashboard to view their clients’ progress, including completion of modules or activities and symptom scores. The dashboard could also be used to recommend modules or activities to clients using the program, and it would be helpful to quickly view their clients’ data before or during a therapy session with them.

### Theme 5: Program is Credible and Trustworthy

#### Theme Overview

This theme emphasizes the importance of a DMHI being credible, reliable, and safe for clients.

#### Evidence-Based and Reputable

Most participants would only recommend DMHIs to their clients if the product had been scientifically evaluated or had demonstrated effectiveness and was developed by organizations or individuals they trusted.

#### Secures and Protects Client Privacy

The DMHI needed to be confidential and protect client data. Participants wanted transparent information about where the client data were stored and who could access it, including in the DMHI. Participants also thought clients should be able to turn clinician access to their data on and off.

### Theme 6: Client Barriers to Using the Program

#### Theme Overview

The final theme refers to barriers that prevent clients from using a DMHI.

#### Program Cost

Most participants believed cost was a barrier to their clients accessing DMHIs and that their clients would want to check the efficacy and suitability of DMHIs before paying for access. Another perspective was that some individuals with mental illness were struggling financially and that DMHIs should be included in treatment costs. Participants also felt uncomfortable with clients paying for DMHIs, considering the already high costs of therapy. However, some acknowledged that cost could indicate to clients that a program has a high level of quality and credibility. Overall, participants emphasized the importance of keeping the costs of DMHIs low.

#### Digital Interventions Not Appropriate for Some Clients

Participants believed that DMHIs would not suit clients who lack access to technology (ie, no mobile phone, computer, or internet access), have poor literacy skills, have cognitive impairments or intellectual disabilities, face psychosocial vulnerabilities (ie, domestic violence and child protection concerns), or require the additional empathy and validation that an in-person therapist can offer.

### Stage 3: Focus Group

#### Demographics

In total, 6 psychologists participated in the 2-part focus group. The mean age was 35 (SD 10; range 26‐54) years. All participants spoke English as their primary language and lived in major Australian cities. Most participants were female (n=5, 83%), and 1 (17%) was male. Overall, 5 (83%) participants were clinical psychologists and 1 (17%) was a provisional psychologist. The participants had worked as mental health professionals for an average of 10 (SD 10; range 1‐28) years and worked in private practice (n=4, 67%), not-for-profit organizations (n=4, 67%), and outpatient settings (n=2, 33%). All psychologists nominated depressive and anxiety disorders as two of the most common mental health issues they treated in their clinical practice. Other common mental health issues treated by participants included trauma and stressor-related disorders (n=5, 83%), personality disorders (n=4, 67%), bipolar and related disorders (n=3, 50%), and obsessive-compulsive and related disorders (n=3, 50%). All psychologists commonly use CBT to treat depression and anxiety. Other treatment modalities included acceptance and commitment therapy (n=4, 67%), mindfulness-based cognitive therapy (n=4, 67%), and schema therapy (n=4, 67%).

#### Thematic Analysis Findings

##### Overview of Findings

Five key themes were identified that related to the resources, support, and training needed to implement blended care into routine care for depression and anxiety: (1) user-friendly resources, training, and support; (2) practical and flexible training; (3) comprehensive implementation support; (4) monitor client progress and risk; and (5) tailor client content and access.

##### Theme 1: User-Friendly Resources, Training, and Support

Psychologists emphasized that resources, support, or training for the blended care system should be user-friendly and facilitate the use of blended care with clients. Psychologists also emphasized that any digital components of a blended care system, such as a web-based dashboard or portal, should be simple, quick, and easy to integrate into their regular clinical practice.

##### Theme 2: Practical and Flexible Training

Psychologists thought training should be ongoing as they used the blended care system and focused on understanding what is included in the program (eg, clinical techniques). Like interviews, the psychologists raised concerns about client confidentiality and how the limitations of duty of care when using DMHIs should be communicated to clients. They suggested that this should be covered in any training provided to mental health professionals for the blended care DMHI. Psychologists also wanted various training resources to be provided, including workshops, written summaries, Frequently Asked Questions, and video-based guides. Receiving professional development credit for an educational module (eg, on blended care) was also considered an important incentive.

##### Theme 3: Comprehensive Implementation Support

The psychologists believed that one-on-one and peer support would be needed to implement blended care. For one-on-one support, they suggested the DMHI support team should regularly check in with mental health professionals using the blended care system and include “Frequently Asked Question” and “Help” sections in a web-based dashboard to provide contact details on how and who to contact for help. Additionally, they recommended facilitating peer support through access to a clinician chat group or message board.

##### Theme 4: Monitor Client Progress and Risk

Psychologists wanted to be able to view and track client progress via a web-based dashboard included in a blended care system. Preferred metrics included questionnaire scores, client responses, and the number of activities completed. Flagging and receiving notifications about at-risk clients based on symptom severity was also essential. Another suggestion was to include a downloadable snapshot of the client’s DMHI use and other data that could be exported to and saved in other practice management software.

##### Theme 5: Tailor Client Content and Access

The psychologists valued the ability to select, schedule, and personalize DMHI content for clients. This included searching for activities with clinical terms (eg, behavioral activation), assigning specific activities to clients, tailoring reminders to suit client needs, and sending content and activity links directly to clients. Having control over the digital “unlinking” of a client during therapy closure or handover to another mental health professional was also important.

## Discussion

### Principal Findings

This was the first participatory design study to investigate Australian mental health professionals’ needs and expectations of a new transdiagnostic, CBT-based blended DMHI that could be integrated into routine care for clients experiencing depression and anxiety. Participants wanted the blended DMHI to be easy to use, flexible to use with clients, and provide a broad range of tailored content to engage and sustain client interest, including evidence-based content from several therapeutic modalities, such as CBT, acceptance and commitment therapy, and dialectical behavior therapy. Participants also provided detailed feedback on the sharing of client data, including a preference for this to occur via a secure email account or web portal, as well as the types of client data that would be most helpful to share. Factors affecting mental health professionals’ acceptance of a blended DMHI, as well as the types of training, support, and resources needed to implement blended care, were also identified. Incorporating features that support the monitoring of client safety into DMHIs and ensuring that DMHIs are free or of low cost for clients could enhance the acceptance of blended care by mental health professionals. Overall, our findings indicate that blended care for depression and anxiety would be feasible and acceptable for Australian mental health professionals to implement, especially psychologists.

### Blended Care Needs and Expectations

Blended care, where a DMHI is integrated with in-person or telehealth sessions, was considered more acceptable than standalone digital interventions. Many of our findings on the needs, preferences, and expectations of mental health professionals for a DMHI that could facilitate blended care align with results from our participatory design study with people with lived and living experiences of depression and anxiety [56] and research with European mental health professionals [28,30,31,38-40]. CBT was the most used therapeutic modality when treating adults with depression and anxiety; however, acceptance and commitment therapy and mindfulness-based cognitive therapy were also common. Given that most participating mental health professionals were experienced CBT-based psychologists, further research will be needed to adapt the blended care system to the needs of other types of mental health professionals. Consistent with the views of people with lived experience [56], participants also expected the blended DMHI to be evidence-based, credible, and protective of client data. These results align with 2 prior blended care studies [28,39] and existing research [22,24,27,60,61] conducted before and during the COVID-19 pandemic, which showed that mental health professionals value DMHIs with strong evidence bases and prioritize client privacy and data security.

A novel finding from this study is mental health professionals’ views on sharing client data, as only 1 blended care study [39] has previously reported this. Behr and colleagues [39] found that psychotherapists believed clients should have control over what data their psychotherapist can see in a DMHI. Our results build upon these findings by providing specific details on how mental health professionals wanted this data sharing to occur. In this study, most participants preferred to receive client information through a secure email account or online portal on a weekly or fortnightly basis and wanted to view data on clients’ progress through the DMHI program, mental health symptoms, and feedback on their treatment. Both our results and those of Behr and colleagues [39] show that mental health professionals value maintaining the security of client data when sharing data with treatment providers. Further research is needed to validate these findings, given the predominance of psychologists in this study and psychotherapists in the Behr and colleagues [39] study. Despite this, the data sharing findings align with qualitative research by Atik and colleagues [28] on blended care, who found that clients were not concerned about sharing personal data within a DMHI, provided their data were used only for treatment purposes. However, these 2 studies [28,39] and our study were conducted during the COVID-19 pandemic; therefore, further replication is needed to ensure that these findings were not due to the unusual circumstances of this worldwide event.

Participants in this study believed blended care could enhance psychotherapy by helping to track a client’s treatment progress and providing clients with psychoeducation and skills practice that support in-session therapy. Similarly, other blended care studies found mental health professionals, who primarily consisted of psychologists and psychotherapists, prefer blended DMHIs that allow clients to track their symptoms and complete homework between therapy sessions [28,29,38,62]. Further, Cerga-Pashoja and colleagues [38] reported that mental health care workers who deliver low-intensity psychological interventions (ie, UK psychological well-being practitioners) wanted blended care to help facilitate continuity between therapy sessions. The current results indirectly support this desire for treatment continuity, as mental health professionals suggested the inclusion of a web-based dashboard that would allow them to recommend program content and review client progress between and before therapy sessions. Relatedly, mental health professionals wanted the blended DMHI to assist with the monitoring of client symptoms and flag if these worsened or indicated suicidality, further contributing to a sense of treatment continuity and mental health support between therapy sessions.

### Blended Care Acceptability

Consistent with previous research [28,39,60] conducted before and during the COVID-19 pandemic, mental health professionals thought that the blended DMHI should direct clients to immediate support if they were feeling unwell or in crisis. Mental health professionals in this study were also wary of using blended care with highly distressed clients or clients with high suicide risk. which is in line with previous research [22,32]. This is despite research evidence indicating that blended care is feasible for treating severe mental illness and may also improve treatment adherence in this population [33]. One reason for the current findings could be that mental health professionals working in private practice, especially psychologists, were well-represented throughout all 3 stages of the participatory design study. These participants may have been more cautious about working with high-risk clients compared to other mental health professionals, as they could be sole traders operating their own private practices or be part of a small business. Other factors influencing participants’ willingness to use DMHIs with clients were client suitability and cost. In line with previous research [22,30], mental health professionals thought blended care might not be appropriate for people with poor digital literacy, limited access to digital technology, or comorbidities that could hamper treatment engagement (eg, cognitive impairment and domestic violence). Prior research also supports our finding that mental health professionals preferred DMHIs to be free or low-cost for clients [26,60]. This highlights the importance of using participatory approaches in DMHI research, especially with practitioners who treat the mental health condition in question, and ensuring sustainable funding models are available for ongoing maintenance and support for blended care systems.

Mental health professionals were also concerned about the time and effort needed to implement blended care with clients. This concern has previously been raised in European studies conducted before the COVID-19 pandemic with CBT-trained mental health professionals working at mental health services that use blended care [30,31,38,40]. However, blended care can also save mental health professionals’ time by allowing the DMHI to cover some aspects of face-to-face therapy, such as psychoeducation, or using the DMHI to continue contact with the client for aftercare and to maintain treatment gains without additional face-to-face therapy sessions [25]. Ensuring these time-saving qualities are realized is key to enhancing the acceptability of blended care models [30,39]. In this study, mental health professionals made several suggestions for minimizing the workload associated with implementing blended care and the types of training and resources that would support them using a blended DMHI with clients. Strategies included trialing the DMHI from the client’s perspective, supplying quick reference guides and resources (eg, written summaries and short videos), and providing a web-based dashboard that could be used to view client progress and prescribe therapeutic content. Mental health professionals also reported a need for ongoing one-on-one and peer support when using blended care with clients and training to help them understand the therapeutic content in the blended DMHI.

### Limitations

This study has several limitations that should be noted. This study occurred during the first and second years of the COVID-19 pandemic (ie, October to December 2020 and July 2021). During this time, the demand for mental health professionals increased while mental health service delivery was disrupted due to quarantining restrictions and physical distancing rules [3,23]. This forced many mental health professionals to switch from face-to-face to telehealth service delivery. It is possible that mental health professionals were more motivated than usual to participate in the research due to the sudden need for many to transition to telehealth service delivery. This may have also influenced our finding that blended care was more acceptable than standalone DMHIs. However, our results do align with a European survey conducted before the COVID-19 pandemic [32], which found that a range of stakeholders involved in depression treatment thought blended care was more acceptable than standalone internet-based treatment for mild, moderate, and severe depression. Further research will be needed to disentangle the effects specific to the COVID-19 pandemic on the blended care system in the future.

The generalizability of our results may also be limited, as the sample was predominantly female and English-speaking and was comprised of individuals who resided in major Australian cities, were psychologists, and worked in private practice within a high-income country. These demographics are consistent with research showing that psychotherapy for mental health treatment within Australia is primarily delivered by psychologists working in private practices [45]. However, further research should consider how the blended care system could be adapted for use by other types of mental health professionals, including those not working in private practice and those in rural, regional, or remote areas of Australia. This could increase the accessibility of blended care for people who may not be able to afford to cover the gap in payments for psychotherapy within the current Australian mental health system. Another limitation of this study is the focus on a transdiagnostic model of care. For highly comorbid mental health diagnoses with shared cognitive, emotional, and biological mechanisms, like depression and anxiety, transdiagnostic approaches have many benefits [7-11]. However, as also mentioned by participants in this study, this approach may not be appropriate for all clients. There is still a need for diagnosis-specific treatment, as some symptoms experienced by a client with a diagnosis, such as bipolar disorder, may not be addressed by a transdiagnostic intervention for depression and anxiety [9].

#### Future Directions

The findings from this study and the participatory design study with people with lived and living experiences of depression and anxiety [56] have been used to create a new Australian blended model of care for depression and anxiety. The present results have been incorporated into a co-design and prototype testing process for a web-based health professional portal that forms part of the myNewWay blended care digital system. The new system also includes a CBT-based and transdiagnostic mobile app for people receiving treatment for depression and anxiety [56]. Phase 2 of the larger Australian project involved refining and evaluating both digital products for use as part of routine care for depression and anxiety. This included an implementation clinical trial with Australian psychologists and their clients. Findings from this clinical trial will inform the development of a workflow model for integrating the new blended care system into a mental health professional’s practice without increasing their workload or compromising quality of care.

### Conclusion

The present findings indicate that Australian mental health professionals who deliver psychotherapy value blended DMHIs that facilitate their clinical practice. Given the similarities between the present findings and the European blended care research [28,30,31,38-40], some elements of optimal blended care may be universal and replicable in mental health care systems in other countries. However, it is important to recognize that blended care will not solve the issue of mental health workforce or funding shortages [3]. Moreover, the increasing incorporation of artificial technology into DMHIs may further complicate the implementation of blended care in the future [17]. Blended care could be an option for leveraging the convenience and accessibility of DMHIs in a way that supports evidence-based treatment and the current workforce while longer-term solutions are sought for these larger system-level issues. Our results, as well as those of others using participatory principles [39,60], further highlight the importance of involving mental health professionals in DMHI design and development. Designing DMHIs that work with, rather than against, existing care pathways for depression and anxiety is crucial for improving access to mental health treatment.

## Supplementary material

10.2196/68789 Multimedia Appendix 1 Web-based survey, interview, and focus group questions.

10.2196/68789 Multimedia Appendix 2 Web-based survey sample comparisons.

10.2196/68789 Multimedia Appendix 3 Use of digital mental health in practice

## Abbreviations

CBT: Cognitive behavioral therapy
DMHI: Digital mental health intervention
RCT: Randomized controlled trial
