# Supplementary material for: Needs and Expectations for the myNewWay Blended Digital and Face-to-Face Psychotherapy Model of Care for Depression and Anxiety (Part 2): Participatory Design Study including Mental Health Professionals
Source: JMIR Hum Factors. 2025 Dec 4;12:e68789. doi: 10.2196/68789 (PMC12677875; doi:10.2196/68789)
Supplement: Multimedia Appendix 1 [file humanfactors-v12-e68789-s001.docx]

## Web-based Survey, Interview and Focus Group Questions

### Web-based Survey Questions

#### Practice Details

- Which of the following best describes your current role? (select all that apply)
  - *Trainee Psychologist*
  - *Counsellor*
  - *Registered Psychologist*
  - *Clinical Psychologist*
  - *General Practitioner*
  - *Psychiatrist*
  - *Mental health nurse*
  - *Social worker*
  - *Other (please specify)*
- What type of setting do you work in? (select all that apply)
  - *Inpatient*
  - *Outpatient*
  - *Private practice*
  - *Not-for-profit organisation*
  - *University*
  - *Other (please specify)*
- How many years have you been working as a Mental Health Professional? (Free text)
- Please choose which mental health issues are most prevalent among your adult clients/patients?
  - *Neurodevelopmental Disorders*
  - *Schizophrenia Spectrum and Other Psychotic Disorders*
  - *Bipolar and Related Disorders*
  - *Depressive Disorders*
  - *Anxiety Disorders*
  - *Obsessive-Compulsive and Related Disorders*
  - *Trauma and Stressor-Related Disorders*
  - *Dissociative Disorders*
  - *Somatic Symptom and Related Disorders*
  - *Feeding and Eating Disorders*
  - *Elimination Disorders*
  - *Sleep-Wake Disorders*
  - *Sexual Dysfunctions*
  - *Gender Dysphoria*
  - *Disruptive, Impulse Control, and Conduct Disorders*
  - *Substance Related and Addictive Disorders*
  - *Neurocognitive Disorders*
  - *Personality Disorders*
  - *Paraphillic Disorders*
  - *Other (please specify)*
- Which treatment modalities do you use most often when working with adults with anxiety and/or depression? Select all that apply.
  - *Cognitive Behavioural Therapy (CBT)*
  - *Acceptance and Commitment Therapy (ACT)*
  - *Interpersonal Psychotherapy (IPT)*
  - *Dialectical Behaviour Therapy (DBT)*
  - *Schema Therapy*
  - *Psychodynamic*
  - *Mindfulness-based cognitive therapy*
  - *Narrative therapy*
  - *Psychoanalysis*
  - *Solution Focused/Brief Therapy*
  - *Eye Movement Desensitization & Reprocessing (EMDR)*
  - *Gestalt*
  - *Humanistic*
  - *Jungian*
  - *Family therapy*
  - *Couple therapy*
  - *Other (please specify)*

#### Digital Mental Health Use

- How often do you use, recommend, or refer adults with anxiety and/or depression to the following digital mental health programs or resources in your work? (Never=1, Sometimes=2, Often=3, Always=4)
  - *Online mental health information and/or psychoeducation sites*
  - *Peer support (e.g., online forums, blogs)*
  - *Mobile phone apps*
  - *Symptom-focused programs (e.g., online CBT programs)*
  - *Online counselling services*
  - *Access services by videoconferencing (e.g., Skype)*
- Which online mental health programs or apps do you currently refer adults with anxiety and/or depression to and why? (Free text)

#### Preferences for Digital Mental Health

- Do you think there is a need for an online mental health program for adults with anxiety and/or depression that integrates into routine care? (i.e., an app or web-browser program that your patients could use together with your clinical sessions?)
  - *Yes*
  - *No*
  - *Not sure*
- In what way would you like an online mental health program for adults with anxiety and/or depression to integrate with your routine care?
  - *As a prequel to therapy (e.g., while patients are on the waitlist)*
  - *Psychoeducation*
  - *Assessments to track symptom change over time (e.g., at commencement, 6 weeks, 3 months)*
  - *To use in session to explain or practice skills (e.g., with examples)*
  - *Be able to refer patient to homework exercises to do in between sessions*
  - *Be able to refer patient to specific modules that support skills they are learning in therapy (e.g., thought challenging)*
  - *Relapse prevention once treatment is done*
  - *To support patients who run out of sessions and cannot afford to pay*
- What would you like to be included in a new online program for adults with anxiety and depression that you could use with your patients/clients? (what types of skills/strategies/exercises?) (Free text)
- What factors would you consider important when determining whether a blended approach is suitable for your clients/patients with depression and/or anxiety (i.e., using an online program together with routine clinical care)? Select all that apply.
  - *Online modules that are relevant to main symptoms/diagnosis of patient*
  - *Absence of severe suicidality*
  - *Absence of psychotic symptoms*
  - *Absence of severe substance use*
  - *Absence of acute medical need that may hinder client/patient's ability to work independently on their treatment*
  - *Intelligence/cognitive functioning of client/patient*
  - *Access to a computer/tablet/phone*
  - *Access to the internet*
  - *Access to a private, safe place to complete program*
  - *Competence in using digital technology (e.g., sufficient internet skills)*
  - *Other (please specify)*
- What type of information from the online program would you want suitable clients/patients to be able to share with you? (Free text)
- What type of information about your client would you like to receive from the online program and at what time intervals? (Free text)
- How would you like information about your client to be shared with you? Select all that apply.
  - *Sent to a secure email account of my choice*
  - *SMS to a nominated phone number*
  - *Secure web portal where I can log-in to see information*
  - *Not sure*
  - *Don't mind*
  - *Other (please specify)*
- Overall, how acceptable do you believe the following treatment options are for your clients with anxiety and/or depression? (Not at all=0, Extremely acceptable=100)
  - Use of standalone digital programs
  - Telehealth sessions
  - Blended approach of using digital programs with face-to-face sessions
  - Blended approach of using digital programs with telehealth sessions

#### Topooco et al. (2017) Questions

1. What do you foresee as the most important challenges for a healthcare professional when integrating digital technology into psychotherapeutic treatment for depression/anxiety? Please select all that apply.
   - *Low clinical effectiveness (psychotherapeutic method needs face-to-face interaction to work)*
   - *Difficulties adapting treatment methods to digital technology*
   - *Not skilled in digital technology*
   - *Degree of accessibility and cost of internet*
   - *Use of digital technology is too time-consuming*
   - *Adherence of the health care professional is low*
   - *Low motivation because of critical attitude towards digital technology*
   - *Other (please specify)*
2. What do you foresee as the most important challenges for a patient when integrating digital technology in a psychotherapeutic treatment for depression/anxiety? Please select all that apply.
   - *Low clinical effectiveness (psychotherapeutic method needs face-to-face interaction to work)*
   - *Difficulties with applying self-help strategies*
   - *Not skilled in digital technology*
   - *Degree of accessibility and cost of internet*
   - *Use of digital technology is too time-consuming*
   - *Low patient adherence*
   - *Low motivation because of a critical attitude towards digital technology*
   - *Other (please specify)*

### Qualitative Interview Questions

- How do you feel in general about online programs or apps for mental health?
  1. *What can be good about them?*
  2. *What can be drawbacks?*
- Could you tell me about your experience with using online programs or apps together with face-to-face therapy?
  1. *What programs or apps have your clients used?*
  2. *What worked well?*
  3. *What did not work? What was missing?*
  4. *{If no experience} Why don’t you use or recommend online programs or apps? What are your main concerns? What would make you recommend them?*
- Do you think there is a need for an online mental health program or app that supports your face to face therapy? Why? Why not?
  1. *If you were going to use an online program or app for adults with anxiety and/or depression to support face-to-face therapy, how would you want to use it? What could it do?*
  2. *What are the most important features to consider? Which of these features are most important to you?*
  3. *What do you want to know about your client over time?*
  4. *How should the program support your clients if they are feeling worse? What level of support do you think is needed?*
  5. *Do you use online software? Would you want the program to integrate with this software? If yes, how?*
  6. *What is the most efficient way for this information to be shared with you/sent to you?*
- How would you know if the online program or app was useful or not useful for your patient?
- Do you have any concerns about using an online program or app to support F2F therapy?
  1. *What situations do you feel you would never use an online program or app with an adult with anxiety/depression?*
  2. *What do you think would make an online program or app engaging for your patients? What would make them come back and use it?*
- I’d now like to talk to you about how an online program could be delivered and promoted.
  1. *What are the main factors that would help you endorse or recommend an online program or app to your clients/patients? What would you look for? What type of information would you need to see? (e.g., proven to work, who created it, who recommended it?)*
  2. *What types of resources/tools would help you to recommend and use an online program or app to your clients/patients? (e.g., video of what is in program and how to use it with clients?)*
  3. *What do you think about programs/apps that you pay for e.g. Headspace?*
  4. *Would your patients pay for an app? Why?/Why not? Who else should pay instead?*
- When did you last make a change in your practice (how you work day to day)? (any type of change).
  1. *What lead to this?*
  2. *What was the outcome and why did you choose this from other options?*
  3. *Were there any unexpected benefits to this change?*
- Do you have any other comments about what the best way is to integrate an online mental health program into routine care?

### Focus Group Questions

- Tell us about a time when you changed your clinical practice (any kind of change) and what led to that change?
- Thinking more specifically about the new blended care service, what would cause or encourage you to implement this service in the first place?
- Do you foresee any risks or challenges in implementing the blended care service into your clinical practice?
  1. *How could these be avoided or overcome?*
- Imagine you're going to start using the new blended care service with your patients tomorrow.

1. *How would you expect this experience to go considering your current practices and systems, etc.?*
2. *How would you go about integrating the new blended care system into practice before, during and after therapy with a patient?*
3. *How are you going to encourage your patients to use the program to support their therapy?*
   - 1. ***Full integration*** *(prescribing combined treatment) to use during each session as part of therapy)*
     2. ***Partial integration*** *(recommend, add-on, adjuncts) to assist homework exercises, consolidation of knowledge, practice skills?*

- What would encourage you to keep using the new blended care service?
- What are the training, professional development and resources that need to sit around the program?

1. *How should this be provided? (who/when/what?)*

- What kind of initial and ongoing support do therapists need?

1. *How should this be provided?*
