# Supplementary material for: Needs and Expectations for the myNewWay Blended Digital and Face-to-Face Psychotherapy Model of Care for Depression and Anxiety (Part 2): Participatory Design Study including Mental Health Professionals
Source: JMIR Hum Factors. 2025 Dec 4;12:e68789. doi: 10.2196/68789 (PMC12677875; doi:10.2196/68789)
Supplement: Multimedia Appendix 2 [file humanfactors-v12-e68789-s003.docx]

## Web-based Survey Sample Comparisons

Statistical comparisons between participants who did and did not complete the full web-based survey were performed using Pearson chi-square (χ^2^) tests of association, Mann-Whitney U-tests or Fisher's exact tests if the χ^2^ test expected cell counts were less than one [1]. The demographics and practice details of participants who did or did not complete the full survey were largely consistent (Table 1). Psychologists were more likely to complete the full survey compared with counsellors who completed the full survey than those who didn’t (Table 1). Participants with work roles in the “other” category were also less likely to complete the full survey. Participants working in private practice were most likely to complete the full survey, while almost half of the participants from not-for-profit settings did not complete the full survey (Table 1).

1. Campbell, I. Chi-squared and Fisher-Irwin tests of two-by-two tables with small sample recommendations. Statistics in Medicine. 2007;26:661–75. PMID: 17315184. doi: 10.1002/sim.2832.

Table 1. Sample characteristics for the web-based survey (*N*=258). Results for years of practice, current role, and current work setting were computed based on data provided by 239 participants who responded to these questions.

|  | Total sample  *N*=258 | Completed  *n*=157 | Incomplete  *n*=101 | Statistic |
| --- | --- | --- | --- | --- |
| **Age (years), *M* (*SD*)** | 47.42 (13.74) | 47.36 (13.65) | 47.52 (13.95) | *U*=8053.00, *P*=.83 |
| **Gender, *n* (*%*)** |  |  |  |  |
| Female | 200 (77.5) | 123 (78.3) | 77 (76.2) | χ^2^(1)=0.16, *P=.*76 |
| Male | 58 (22.5) | 34 (21.7) | 24 (23.8) | - |
| **English primary language at home, *n* (*%*)** | 227 (88) | 141 (89.8) | 86 (85.1) | χ^2^(1)=1.26, *P*=.33 |
| **First Nation Australian Origin,**  ***n* (*%*)** | 4 (1.6) | 1 (0.6) | 3 (3) | Fisher’s exact, *P*=.30 |
| **Residence in Australia, *n* (*%*)** |  |  |  |  |
| Regional or remote | 62 (26.2) | 38 (25.3) | 24 (27.6) | χ^2^(1)=0.15, *P=.*76 |
| Major cities | 175 (73.8) | 112 (74.7) | 63 (72.4) | - |
| **Years of practice, *M* (*SD*)** | 15.08 (11.89) | 15.57 (12.02) | 14.13 (11.65) | *U*=6011.50, *P*=.40 |
| **Current role^a^**, ***n* (*%*)** |  |  |  |  |
| Registered Psychologist | 45 (18.8) | 37 (23.6) | 8 (9.8) | χ^2^(1)=6.72, *P*=.01 |
| General Practitioner | 43 (18) | 31 (19.7) | 12 (14.6) | χ^2^(1)=0.95, *P*=.38 |
| Clinical Psychologist | 37 (15.5) | 29 (18.5) | 8 (9.8) | χ^2^(1)=3.13, *P*=.09 |
| Social Worker | 28 (11.7) | 15 (9.6) | 13 (15.9) | χ^2^(1)=2.07, *P*=.20 |
| Mental Health Nurse | 26 (10.9) | 17 (10.8) | 9 (11) | χ^2^(1)=0.00, *P*=1.00 |
| Counsellor | 20 (8.4) | 8 (5.1) | 12 (14.6) | χ^2^(1)=6.39, *P*=.02 |
| Trainee Psychologist | 11 (4.6) | 9 (5.7) | 2 (2.4) | Fisher’s exact, *P*=.34 |
| Psychiatrist | 5 (2.1) | 4 (2.5) | 1 (1.2) | Fisher’s exact, *P*=.66 |
| Other current role^b^ | 36 (15.1) | 15 (9.6) | 21 (25.6) | χ^2^(1)=10.85, *P*=.001 |
| **Current work setting^a^, *n* (*%*)** |  |  |  |  |
| Private Practice | 119 (49.8) | 87 (55.4) | 32 (39) | χ^2^(1)=5.79, *P*=.02 |
| Not-for-profit organization | 49 (20.5) | 25 (15.9) | 24 (29.3) | χ^2^(1)=5.89, *P*=.02 |
| Outpatient | 38 (15.9) | 26 (16.6) | 12 (14.6) | χ^2^(1)=0.15, *P*=.72 |
| Inpatient | 28 (11.7) | 19 (12.1) | 9 (11) | χ^2^(1)=0.07, *P*=.84 |
| University | 25 (10.5) | 17 (10.8) | 8 (9.8) | χ^2^(1)=0.07, *P*=.83 |
| Other work setting^c^ | 29 (12.1) | 15 (9.6) | 14 (17.1) | χ^2^(1)=2.86, *P*=.10 |
